# Eco-Friendly Biomass Production and Identification of Active Compounds of *Paenibacillus polymyxa* EB.KN35 with Potent Anti-*Fusarium oxysporum* Effect

**DOI:** 10.3390/microorganisms13040800

**Published:** 2025-03-31

**Authors:** Van Anh Ngo, Anh Dzung Nguyen, San-Lang Wang, Tu Quy Phan, Thi Ha Trang Tran, Dinh Sy Nguyen, Van Bon Nguyen

**Affiliations:** 1 Institute of Biotechnology and Environment, Tay Nguyen University, Buon Ma Thuot 630000, Vietnam; nvanh@ttn.edu.vn (V.A.N.); nadzung@ttn.edu.vn (A.D.N.); tthatrang@ttn.edu.vn (T.H.T.T.); 2 Department of Chemistry, Tamkang University, New Taipei City 25137, Taiwan; 3 Department of Science and Technology, Tay Nguyen University, Buon Ma Thuot 630000, Vietnam; phantuquy@ttn.edu.vn (T.Q.P.); ndsy@ttn.edu.vn (D.S.N.)

**Keywords:** *Fusarium oxysporum*, *Paenibacillus polymyxa*, bioactive compounds, agricultural byproduct reuse, bioreactor, trypsin

## Abstract

*Fusarium oxysporum* is a fungal plant pathogen for over 100 agricultural crop species. There are strategies for managing *Fusarium* wilt, including antagonistic bacteria that offer a promising and sustainable effect. In this work, among the various endophytic bacterial strains, *Paenibacillus polymyxa* EB.KN35 was selected as the best antifungal strain against *F. oxysporum*. For eco-friendly biomass production of this bacterium, some agricultural byproducts were tested for cultivation, and a soybean processing byproduct (SPBP) was found to be a suitable C/N source for *P. polymyxa* EB.KN35 fermentation. The utilization of a 14 L bioreactor system for *P. polymyxa* EB.KN35 fermentation achieved a high biomass productivity (3.46 × 10^11^ CFU/mL) in a short time (8 h). In bioactive compound analysis, EB.KN35 was found to be secreting several plant growth-promoting compounds such as GA3, IAA, kinetin, and zeatin (via HPLC) and eleven volatile compounds (via GC–MS). The docking study indicated that some volatile compounds (1, 2, 4, and 9) may play a significant role in inhibiting *F. oxysporum*. The study results highlight the potential for reusing a soybean processing byproduct as a C/N source for the bioproduction of *P. polymyxa* EB.KN35 with potential use as a biocontrol agent and biofertilizer.

## 1. Introduction

*Fusarium oxysporum* is a soil-borne ascomycete fungus ranked fifth among the most destructive plant pathogenic fungi worldwide and seriously harms more than 100 major agricultural crop species [1,2,3]. *F. oxysporum* produces chlamydospores that can survive in infected soil for decades [4]. Current strategies for managing *Fusarium* wilt include cultural practices, chemical treatments, and biological control. Among these, microbial biocontrol agents, such as antagonistic bacteria, offer a promising and sustainable alternative [5,6,7,8,9,10]. Beneficial microbes have various defense mechanisms against pathogens, including direct antagonism, indirect inhibition of pathogens through secondary compounds, antibiotics, or cell wall-degrading enzymes, as well as competition with pathogens [11,12]. In addition, beneficial bacteria also help stimulate plant growth and enhance the sustainability of agroecosystems [13,14].

*Paenibacillus polymyxa* is an effective biocontrol agent for sustainable crop production. *P. polymyxa* has been proven to be an effective fungicidal bioagent against various pathogens such as *Verticillium longisporum*, *Fusarium* sp., *Ralstonia solanacearum*, etc. [15,16,17,18,19,20,21,22,23,24]. Notably, *P. polymyxa* has strong potential against *F. oxysporum*, causing diseases in watermelon, cucumber, rape, *Cryptomeria fortunei*, and *Urtica dioica* [17,18,19,20,21,22,23,24]. However, its potential against *F. oxysporum* disease in durian plants has not been recorded. *P. polymyxa* has gained interest in mass production via fermentation for application. Almost all the previous studies used commercial media for cultivation. For cost-effective production, some byproducts have been used in *P. polymyxa* fermentation, such as sweet potato starch wastewater, molasses, pear bagasse, wheat bran, etc. [25,26,27,28,29,30,31,32,33,34,35]. In recent years, soybean flour has been used in *P. polymyxa* fermentation. However, the reuse potential of its byproduct has not been considered [33]. In particular, assessing some common byproducts such as soybean processing byproducts, rice bran processing byproducts, and cassava processing byproducts has not been reported. In addition, almost all the previous works [25,26,27,28,29,30,31,32,33,34,35] reported biomass production on a small scale using flasks. The bioactive compounds produced by this bacterial strain and its mechanism of interaction with *F. oxysporum* trypsin are also not well-studied.

Protein trypsin-like proteases (1TRY) targeting *F. oxysporum*, also known as triosephosphate isomerase, are crucial metabolic enzymes involved in glycolysis and gluconeogenesis. In *Fusarium oxysporum*, 1TRY is an important enzyme with multiple functions. It is a key metabolic enzyme, and it also contributes to fungal virulence. In fungal pathogenicity, 1TRY plays a role in immune evasion, oxidative stress resistance, host cell interaction, and host adaptation. These abilities make 1TRY a promising target for antifungal drug development [36,37,38].

In this study, among the various tested endophytic bacterial strains, *P. polymyxa* EB.KN35 was screened as the most promising strain for the biocontrol of *F. oxysporum*. Regarding the eco-friendly production of this potential strain, various agricultural processing byproducts were used as C/N sources for fermentation. *P. polymyxa* EB.KN35 was further tested for fermentation on a larger scale using a 14 L bioreactor system. In addition, the bioactive compounds produced by *P. polymyxa* EB.KN35 were identified using GC–MS and HPLC. The molecular docking study and DPT calculation were also conducted to study the interaction mechanism of bioactive compounds with protein 1TRY, targeting *F. oxysporum*.

## 2. Materials and Methods

### 2.1. Materials

The pathogenic fungus *Fusarium oxysporum* and 110 endophytic bacterial strains were stored at the Institute of Biotechnology and Environment—Tay Nguyen University, Vietnam. PDA medium was used for fungal activation, and LB medium was used for bacterial activation at 28 °C. Soybean processing byproducts (SPBPs), rice bran processing byproducts (RBPBPs), and cassava processing byproducts (CPBPs) were collected in Buon Ma Thuot, Dak Lak, Vietnam.

### 2.2. Methods

#### 2.2.1. Endophytic Bacterial Strains Screening

The antifungal activity of all the endophytic bacterial strains was evaluated according to the method of Ngo et al. [39]. According to this method, the pathogenic fungus *F. oxysporum* was placed in the middle of a Petri dish, the antagonistic endophytic bacteria were inoculated 3 cm away from the pathogenic fungus with a 1 cm bacterial inoculation line, the activity evaluation medium was PDA medium, and each experiment was repeated 3 times. The experimental plates were incubated at 30 °C until the front edge of the *F. oxysporum* fungus in the control group reached the edge of the plate. For the control group, only the pathogenic fungus was inoculated on the plate.

**Figure 1:**
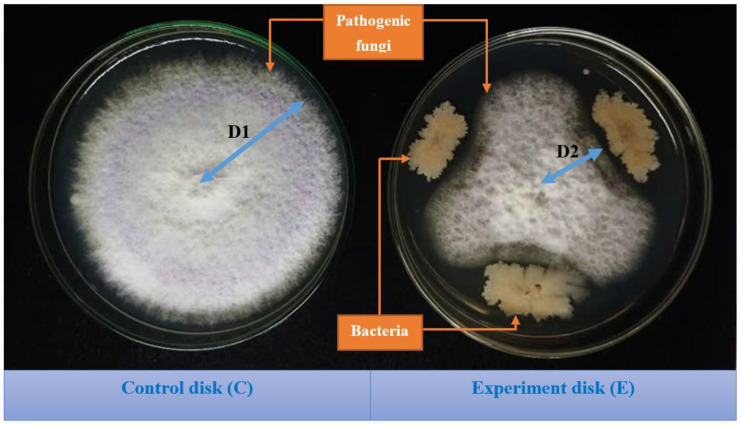
Images of the antifungal assays of the durian endophytic bacterial strain. In the control dish (C), the pathogenic fungus was grown on potato dextrose agar (PDA), and in the experiment dish (T), also on PDA, the pathogenic fungus was treated with an endophytic bacterial strain. After five days of cultivation, the radii (cm) of the fungal mycelial spread on the control dish (D1) and the experimental dish (D2) were measured for the calculation of antifungal activity.

The growth inhibition activity was calculated as follows:

The inhibition rate of mycelial growth (%) = [(D1 − D2)] / D1] × 100, where D1 = radius of the mycelium growing on the control plate (cm) and D2 = radius of the mycelium growing on the plate cocultured with bacteria (cm). Figure 1 illustrates the measuring radius of the mycelium.

#### 2.2.2. Method for Assessing Bacterial Density After Culture

Microbial density was assessed using the colony counting method on agar plates [40].

#### 2.2.3. Fermentation Optimization

To achieve high biomass production of *P. polymyxa* EB.KN35, C/N sources, types of mineral salts, and some parameters were examined.

♦ The effect of C/N sources on the growth of *P. polymyxa* EB.KN35

Various agricultural processing byproducts, including 1.5% SPBP, 1.5% RBPBP, and 1.5% CPBP, were supplemented into the medium (30 mL) containing 0.5% NaCl, with the initial pH of 7, a shaking speed of 150 rpm for 24 h (*), and the control medium LB (1% peptone, 0.5% yeast extract, and 0.5% NaCl). The fermentation condition was symbolized as (*) and was further used for some experiments. Then, the bacterial density after culture was determined according to the method of Mai et al. [40] to find the most promising substrate.

♦ The effect of agricultural processing byproducts (SPBPs) and LB ratio on the growth of *P. polymyxa* EB.KN35

SPBP and LB were mixed at various ratios of 0/10, 3/7, 5/5, 7/3, and 10/0 with pH = 7 and used as a C/N source for *P. polymyxa* EB.KN35 fermentation. The ratios were tested as follows:

+Test 1: 1.5% LB

+Test 2: 0.45% SPBP+1.05% LB

+Test 3: 0.75% SPBP+0.75% LB

+Test 4: 1.05% SPBP+0.45% LB

+Test 5: 1.5% SPBP+0.5% NaCl

*P. polymyxa* EB.KN35 was cultivated under the conditions mentioned above (*). The bacterial density after culture was determined according to Section 2.2.2 to identify the most suitable substrate ratio.

♦ The effect of C/N source concentration on the growth of *P. polymyxa* EB.KN35

Several concentrations of the 7SPBP/3LB (1%, 1.25%, 1.5%, 1.75%, 2%, 2.25%) were added to the liquid medium containing 0.5% NaCl under the abovementioned conditions (*). From the density results after culture, the optimal substrate concentration was identified for the next experiments.

♦ The effect of mineral salts on the growth of *P. polymyxa* EB.KN35

The influence of some types of mineral salts on the growth of *P. polymyxa* EB.KN35 was determined in the previous studies [25,31,35]. In this study, some other sources of sulfate and phosphate salts were investigated:

The influence of some types of sulfate salts was investigated: MgSO_4_, CaSO_4_, MnSO_4_, ZnSO_4_, and FeSO_4_. The culture medium (30 mL) contained 1.75% C/N source, 0.05% sulfate salt, and the control medium (with 0.5% NaCl instead of 0.05% sulfate salt), with an initial pH = 7 and 150 rpm shaking speed for 24 h. Then, the suitable sulfate salts were determined according to Section 2.2.2 and selected for further tests.

The influence of some types of phosphate salts was investigated at 0.1% (K_2_HPO_4_, KH_2_PO_4_, Na_2_HPO_4_, Ca_3_(PO_4_)_2_), as well as 0.05% FeSO_4_. The culture medium (30 mL) contained 1.75% C/N source, 0.05% sulfate salt, and the control medium (with 0.5% NaCl), with an initial pH = 7 and 150 rpm shaking speed for 24 h. This allowed for the determination of the most suitable phosphate salt according to Section 2.2.2.

♦ The effect of some fermentation condition parameters on the growth of *P. polymyxa* EB.KN35

Some other parameters that affect the growth of *P. polymyxa* EB.KN35 during fermentation was also studied according to the previous reports [25,26,27,28,29,30,31,32,33,34,35], including the initial pH of the culture medium (6.25, 6.5, 6.75, 7.0, 7.25, and 7.5), temperature (25 °C, 28 °C, 30 °C, 32 °C, and 34 °C), and culture time (16, 18, 20, 22, 24, and 26 h). The culture medium (30 mL) contained 1.75% C/N source, 0.1% KH_2_PO_4_, and 0.05% FeSO_4_, with a 150 rpm shaking speed for 24 h, and bacterial density was determined after culture according to Section 2.2.2. The culture time experiment involved 30 mL of a medium containing 1.75% C/N source, 0.1% KH_2_PO_4_, and 0.05% FeSO_4_, with a 150 rpm shaking speed, and bacterial density was determined after 16 h, 18 h, 20 h, 22 h, 24 h, and 26 h (method from Section 2.2.2.).

♦ Scaling up the biomass production of *P. polymyxa* EB.KN35 using a 14 L bioreactor system

A 14 L bioreactor system (BioFlo/CelliGen 115, New Brunswick, NJ, USA) contained 7.2 L of a medium including 1.75% C/N source, 0.1% KH_2_PO_4_, 0.05% FeSO_4_, with the parameters of the system adjusted to match the results of experiment 3.2 (30 °C, dissolved oxygen at 15 vvm, stirring speed at 300 rpm). After that, 0.8 L of bacterial culture were added to the bioreactor, and the microbial density was monitored at 2 h, 4 h, 6 h, 8 h, and 10 h after culture (CFU/mL).

#### 2.2.4. Detection and Identification of Bioactive Compounds Biosynthesized by *P. polymyxa* EB.KN35

HPLC was used to determine the growth-promoting compounds, including IAA, GA3, kinetin, and zeatin according to the method presented by Nguyen et al. [41]. Besides, the volatile compounds of this strain were identified through the GC/MS system (including ITQ 900 Mass Spec-Thermo and GC-Thermo Trace GC Ultra, Thermo Fisher Scientific, headquartered in Waltham, MA, USA). The TG-SQC capillary column (30 m × 0.25 mm × 0.25 μm) was filled with carrier gas helium (99.99%) at a constant flow rate of 1 mL/min. The bacterially cultivated broth was dissolved in methanol. One μL of the sample solution was injected with a 10:1 split ratio at a 250 °C injector temperature and constant ion source temperature of 230 °C. Initially, the column was kept at 50 °C for 2 min, then increased to 250 °C at a speed of 10 °C/min, and further increased to 280 °C for 10 min. The MS data were collected at the conditions of 70 eV of electron energy, 0.5 s of scanning interval time, and the range of fragments from 35 to 650 Da and were then compared to the standard compounds in the Mass Spectra Library (NIST 17 and Wiley) for identification.

#### 2.2.5. Molecular Docking and DFT Calculation

Molecular docking was performed following the protocols presented in the previous report [42]. The structure of the protein 1TRY targeting *F. oxysporum* was obtained from the RCSB Protein Data Bank, and its 3D structure was optimized (at a virtual pH of 7) using the MOE-2015.10 software. The most active binding sites on the protein 1TRY were searched using the site-finding function of the MOE-2015.10 software. The structure of the ligands (volatile compounds produced by *P. polymyxa* EB.KN35) was also prepared using the MOE software with the following optimization parameters: force field MMFF94x; R-field 1: 80; cutoff, rigid water molecules, space group p1, cell size (10, 10, 10); cell shape (90, 90, 90); and gradient 0.01 RMS kcal·mol^−1^A^−2^. All the ligands were docked into the binding sites of the protein 1TRY, and the data were obtained from the docking performance at the most active binding site. Some output data, including root mean square deviation (RMSD), docking score (DS), linkage type, distances between linkages, and amino acid compositions, were obtained for analysis. The highest-energy molecular orbital (HOMO) and the lowest unoccupied molecular orbital (LUMO) of these ligands were examined using density functional theory (DFT) at the B3LYP/6-31G theoretical level.

#### 2.2.6. Statistical Analysis 

The results of the experiments in Section 3.1 and Section 3.3 were processed using the Microsoft Office Excel software, and the experiment in Section 3.2 employed ANOVA using the SPSS 22.0 software. All the experiments were randomly designed. The data represent the average value of 3 repetitions ± standard deviation with a significance level of *p* < 0.05.

## 3. Results and Discussion

### 3.1. Screening of Endophytic Bacterial Strains with Potent Fungicidal Efficacy Against Fusarium oxysporum

**Figure 2:**
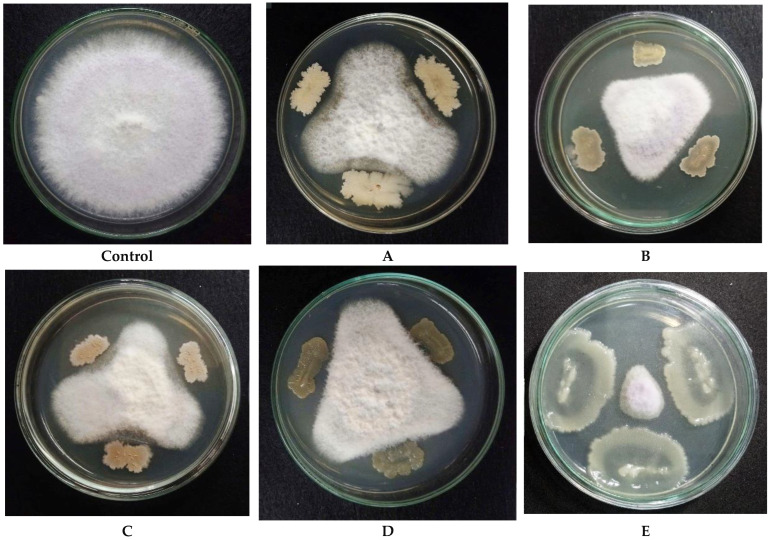
Evaluation of the antifungal potential of 5 endophytic strains against the pathogenic fungus *F. oxysporum* isolated in the Central Highlands, Vietnam. **Control** is the control plate and (**A**) *B. amyloliquefaciens* EB.CK9, (**B**) *B. amyloliquefaciens* EB.EH18, (**C**) *B. methylotrophicus* EB.EH34, (**D**) *B. siamensis* EB.KN10, and (**E**) *P. polymyxa* EB.KN35.

A total of 110 endophytic bacterial strains were evaluated for their antifungal effect against *F. oxysporum*. As shown in Table A1, fifty-two strains showed mycelial growth inhibition, with inhibition values in the range of 11.79–79.58%, and five strains were found to show significant mycelial growth inhibition (≥50.83%). Of these, the endophytic strains *Bacillus amyloliquefaciens* EB.CK9, *Bacillus amyloliquefaciens* EB.EH18, *Bacillus methylotrophicus* EB.EH34, and *Bacillus siamensis* EB.KN10 showed moderate inhibition values of 50.83%, 56.67%, 55.83%, and 51.25%, respectively. *Paenibacillus polymyxa* EB.KN35 was demonstrated to be the most effective antifungal strain with the highest inhibition value of 79.58%. The inhibition images of these five strains against *F. oxysporum* on Petri dishes are illustrated in Figure 2.

*Paenibacillus polymyxa* is an effective biological control agent against various pathogens such as bacteria, root-knot nematodes, and some fungi and oomycetes (*Verticillium longisporum*, *Fusarium* sp., *Ralstonia solanacearum*) through various mechanisms [15,16,17,18,19,20,21,22,23,24]. *P. polymyxa* is also recognized as a potential biocontrol agent against *F. oxysporum* [17,18,19,20,21,22,23,24]. Some endophytic bacteria, such as *P. polymyxa*, isolated from healthy plants, have been shown to control *Fusarium* wilt on various crops. In addition, they have beneficial effects on promoting plant growth under field conditions [15,16]. Similarly, *P. polymyxa* PJH16 isolated from cucumber soil and *P. polymyxa* NSY50 isolated from vinegar waste compost have been identified as biocontrol agents effective in controlling *Fusarium* wilt, which is a major soil-borne disease in cucumber [20,24]. In this study, we noted that the durian endophytic *P. polymyxa* EB.KN35 exhibited a strong antagonistic potential against *F. oxysporum*, a pathogenic durian isolate from the Central Highlands of Vietnam. For the first time, durian endophytic bacterium *P. polymyxa* has been recorded as effective against *F. oxysporum* (79.58%). The potential biocontrol of *P. polymyxa* against *F. oxysporum* in various crops from the literature review is summarized in Table 1. *P. polymyxa* showed mycelial growth inhibition against *F. oxysporum*, with inhibition values in the range of 36–88.36%. Thus, the anti-*F. oxysporum* activity of *P. polymyxa* EB.KN35 recorded in this work is comparable to that of the previous studies.

### 3.2. The Effects of the Substrate, Salt, and Some Fermentation Parameters on the Growth of P. polymyxa EB.KN35

Currently, *P. polymyxa* is being widely studied, mainly for fermentation in commercial media. There have been some reports on cultivating *P. polymyxa* using byproducts (wastewater from sweet potato starch, molasses, pear pulp, wheat bran, etc.) to reduce costs and create environmentally friendly products [25,26,27,28,29,30,31,32,33,34,35]. Consequently, this study investigates the possibility of using agricultural byproducts for *P. polymyxa* EB.KN35 fermentation to achieve high productivity in its biomass and bioactive compounds.

#### 3.2.1. The Effect of the Substrate Source on the Growth of *P. polymyxa* EB.KN35

**Figure 3:**
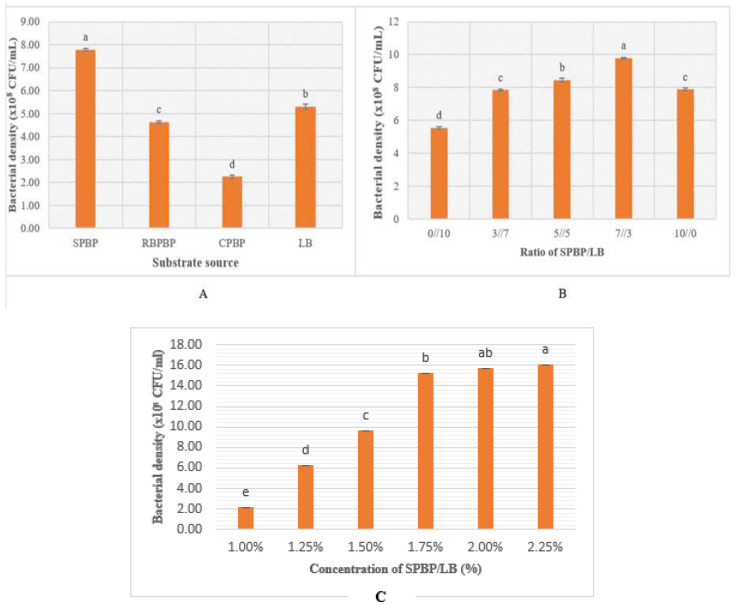
The effect of the substrate source on the fermentation of the endophytic bacterial strain *P. polymyxa* EB.KN35. (**A**) The effect of four substrate sources, including soybean processing byproducts (SPBPs), rice bran processing byproducts (RBPBPs), cassava processing byproducts (CPBPs), and Luria broth (LB) used at a concentration of 1.5%. (**B**) The effect of different ratios of agricultural processing byproducts (SPBPs) and LB. (**C**) The effect of the different concentrations of the substrate (SPBPs/LB = 7/3). Means followed by different letters (a, b, c) are significantly different according to Duncan’s Multiple Range Test at *p* < 0.05.

*P. polymyxa* EB.KN35 strain was fermented using several agricultural processing byproducts (RBPBPs, SPBPs, and CPBPs) as C/N sources for cultivation. As shown in Figure 3A, the density of *P. polymyxa* EB.KN35 grows strongly in SPBPs, reaching 7.8 × 10^8^ CFU/mL. The result of this study is different from the previous reports. Kim et al. [26] used TSB in the cultivation of *P. polymyxa* T5 and gained 10^6^ CFU/mL. Meanwhile, *P. polymyxa* BY-28 reuses wastewater from sweet potato starch, reaching 9.7 × 10^9^ CFU/mL; *P. polymyxa* GBR-1 uses commercial medium BHI, reaching 4.6 × 10^9^ CFU/mL; and *P. polymyxa* D1 reuses pear pulp and wheat bran, reaching 4.52 × 10^9^ CFU/mL [29,30,31]. In addition, skimmed milk and molasses were used as C/N sources in the fermentation of *P. polymyxa* Kp10 and *P. polymyxa* IN937a, reaching a density of 10^8^ CFU/mL and 6.606 × 10^9^ CFU/mL, respectively [33,35]. Soybean processing byproducts (SPBPs) have high nutritional content, including about 50% crude oil, 25% proteins, 10% lipids, and other nutrients [43]. Therefore, SPBPs are a suitable substrate for the fermentation of the endophytic bacterium *P. polymyxa* EB.KN35, and it was selected for further research. Notably, this is the first record of the ability to reuse these three agricultural processing byproducts for the *P. polymyxa* strain cultivation.

The different ratios of SPBPs and LB had a clear impact on the fermentation of *P. polymyxa* EB.KN35. The results in Figure 3B indicate the best growth of *P. polymyxa* EB.KN35 was recorded at the SPBPs/LB ratio of 7/3, with bacterial densities reaching a high of 9.78 × 10^8^ CFU/mL. Thus, this ratio was chosen for subsequent experiments to use soybean processing byproducts. The C/N source (SPBPs/LB) concentration was also assessed for the effect on bacterial biomass production. The experimental results illustrated in Figure 3C show that the different concentrations of SPBPs/LB (0.5–2.5%) were directly proportional to the growth of *P. polymyxa* EB.KN35, with SPBPs/LB concentrations of 1.75–2.5% ( 7:3 ratio) showing the highest growth. Therefore, we used the SPBPs/LB concentration of 1.75% ( 7:3 ratio) for the following experiments to save production materials.

#### 3.2.2. The Effect of Mineral Salts and Some Fermentation Parameters

**Figure 4:**
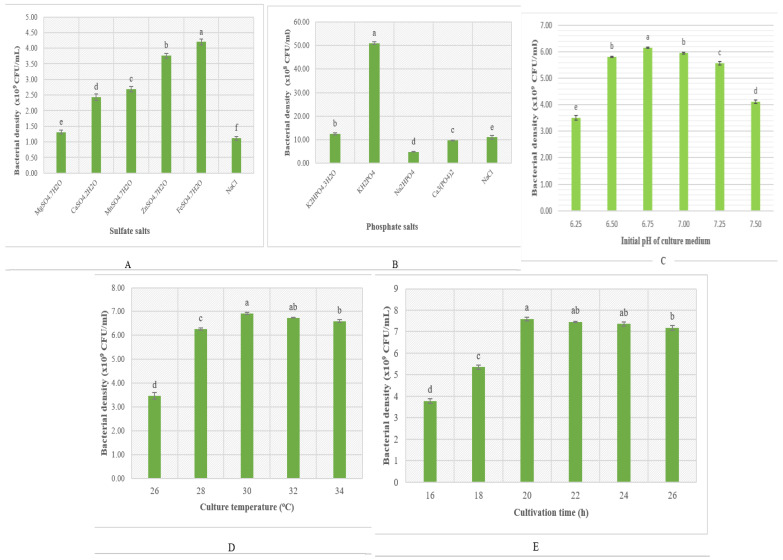
The effect of mineral salts and fermentation parameters on the cell growth of *P. polymyxa* EB.KN35 using a 1.75% SPBPs/LB substrate (7:3 ratio). (**A**) The effect of sulfate salts tested at the concentration of 0.05% (**B**). The effect of phosphate salts tested at a concentration of 0.01%. (**C**) The effect of the initial pH of the culture medium. (**D**) The effect of cultivation temperature. (**E**) The effect of cultivation time. Means followed by different letters (a, b, c) are significantly different according to Duncan’s Multiple Range Test at *p* < 0.05.

Sulfate and phosphate salts have been reported to enhance bacterial fermentation by achieving higher productivity of biomass and bioactive compounds in numerous works [44,45]. Thus, in this study, the effect of sulfate and phosphate salts on bacterial growth was tested, and the results are presented in Figure 4A and Figure 4B, respectively. As shown in Figure 4A, all sulfate salts were found to promote bacterial biomass growth more than the original environment. Among them, *P. polymyxa* EB.KN35 showed the highest growth in the medium supplemented with FeSO_4_, reaching a cell density of 4.12 × 10^9^ CFU/mL. Meanwhile, three out of the four added phosphate salts (K_2_HPO_4_, Na_2_HPO_4_, and Ca_3_(PO_4_)_2_) were ineffective for the growth of strain EB.KN35 with the bacterial density equal to or even lower than in the original environment (Figure 4B). Notably, supplementation with KH_2_PO_4_ strongly promoted the development of *P. polymyxa* EB.KN35, with a significantly high bacterial density of 5.06 × 10^9^ CFU/mL.

Some culture conditions, such as the initial pH of the culture medium, cultivation temperature, and cultivation time, were also examined for their effect on bacterial biomass production, and the results are shown in Figure 4C, Figure 4D, and Figure 4E, respectively. Overall, *P. polymyxa* EB.KN35 grew best using a 1.75% SPBPs/LB substrate (7:3 ratio), 0.05% FeSO_4_, and 0.1% KH_2_PO_4_ with pH = 6.75 at 30 °C in 20 h of culture. This work reports the reuse of SPBPs as a new low-cost and effective substrate for the growth of *P. polymyxa* EB.KN35. The results of this study concerning the effect of temperature and the initial pH of medium cultivation are similar to those of the previous reports. In the report by Gong et al. [25], *P. polymyxa* BY-28 fermented using bean cake powder at 30 °C reached 3.1–3.3 × 10^8^ CFU/mL. *P. polymyxa* M-1 fermented using GSC at 30 °C reached 4 × 10^7^ CFU/mL [27]. Similarly, *P. polymyxa* reached 10^5^–10^7^ CFU/mL when cultured at 25–30 °C [28]. Gao et al. [32] reported that *P. polymyxa* Pp-7250 produced a biomass of 10^7^ CFU/mL at 30 °C, pH 6–8, with wheat starch and soybean flour as the main substrate sources, while *P. polymyxa* NSY50 reached a density of 2.5 × 10^8^ CFU /mL at 28 °C using LB medium [20]. However, the growth of *P. polymyxa* on the substrate of soybean byproducts combined with FeSO_4_ and KH_2_PO_4_ achieving high biomass in a short time (20 h) is a new finding of this work. In summary, the growth of *P. polymyxa* strains depends on the substrate and the bacterial strain.

### 3.3. Scaling up Biomass Production of P. polymyxa EB.KN35 via Fermentation in a 14 L Bioreactor

**Figure 5:**
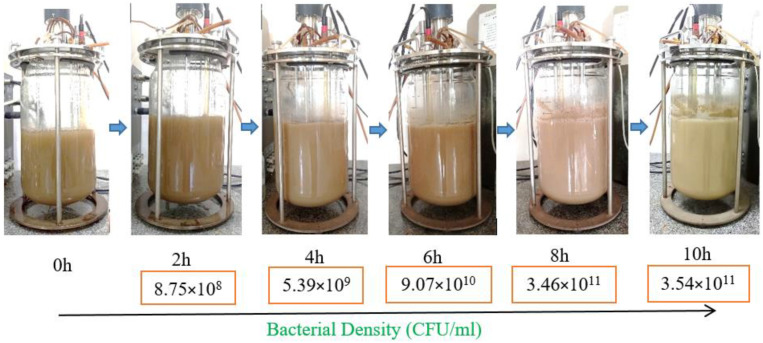
The results of P. polymyxa EB.KN35 biomass production in a larger fermentation system.

For enhancing *P. polymyxa* EB.KN35 biomass productivity and shortening the fermentation time, a 14 L bioreactor system was applied for fermentation from 0 h to 10 h in this experiment. Monitoring the fermentation process every 2 h, the data were recorded and illustrated in Figure 5. Accordingly, the bacterial density increased gradually over time and reached the optimum after 8 h of fermentation, with a maximum density of 3.46 × 10^11^ CFU/mL. Thus, compared to fermentation at the small flask scale (Figure 4E), scaling up to a 14 L bioreactor reduced the fermentation time from 20 h to 8 h while increasing biomass yield.

In an earlier report by Kaziūnienė et al. [34], *P. polymyxa* MVY-024 was cultivated in an EDF-5.4 bioreactor (with the working volume of up to 4.5 L out of the total volume of 6.2 L) using yeast extract and molasses to reach a density of 2.2 × 10^9^ CFU/mL^−1^. The research on *P. polymyxa* fermentation on a large scale has been rarely reported. To our current knowledge, this is the first work to report the fermentation of *P. polymyxa* using a 14 L bioreactor system with a working volume of 8 L. In addition, the study demonstrates the use of SPBPs in the fermentation of increased biomass of *P. polymyxa* strain EB.KN35 in a low-cost, safe, effective, and environmentally friendly manner.

### 3.4. The Bioactive Compounds Produced by P. polymyxa EB.KN35

*P. polymyxa* has been reported to promote plant growth by producing a variety of phytohormones such as auxins, cytokinins, indole-3-acetic acid (IAA), etc., and its fungicidal effect is closely related to the production of volatile compounds (VOCs) [46,47,48]. For a better understanding of the plant-promoting effect and fungicidal activity of *P. polymyxa* EB.KN35, we used HPLC and GC–MS to identify the bioactive compounds produced by *P. polymyxa* EB.KN35.

**Figure 6:**
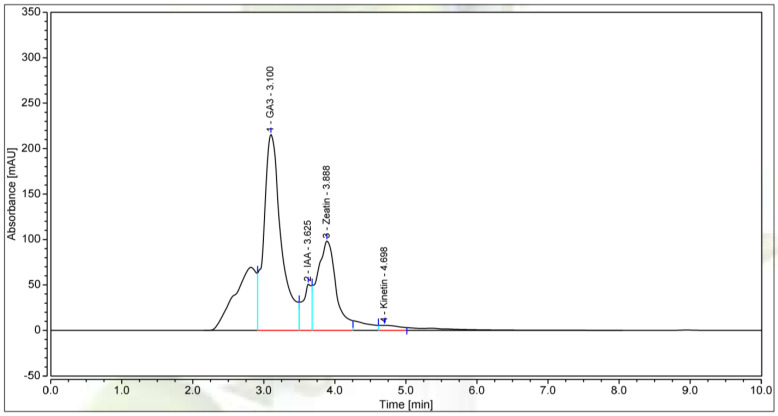
The HPLC profiles of plant growth-promoting compounds biosynthesized by *P. polymyxa* EB.KN35. The bacterial strains were cultivated in King’s B medium at 28 °C. After 2 days, the fermentation broth was collected and centrifuged at 6000 rpm/10 min, and the residue was removed. The supernatant was filtered through a 0.45 µm membrane and used for the analysis of plant growth stimulants by the HPLC system. Five µL of the culture filtrate were injected into the HPLC system and separated using a C18 column before detecting PGPCs at 254 nm.

HPLC fingerprinting shown in Figure 6 indicated that *P. polymyxa* EB.KN35 produced 4 plant growth-promoting compounds, including GA3, zeatin, IAA, and kinetin. Of these, GA3 and zeatin were produced at a high-level yield in the culture medium with concentrations of 1168 µg/mL and 38.78 µg/mL, while IAA and kinetin were produced in minor amounts. The report by Tokmakova et al. [49] recorded that another *P. polymyxa* KB strain also produced these four growth stimulants with a content of 2.48–81.15 μg/g dry biomass. Additionally, some reports have recorded the IAA production activity of other *P. polymyxa* strains such as *P. polymyxa* SK1 and *P. polymyxa* SC2 producing IAA at concentrations from 13 to 31 μg/mL and *P. polymyxa* ZYPP18 producing IAA at a concentration of 3050 μg/mL [50,51,52].

**Figure 7:**
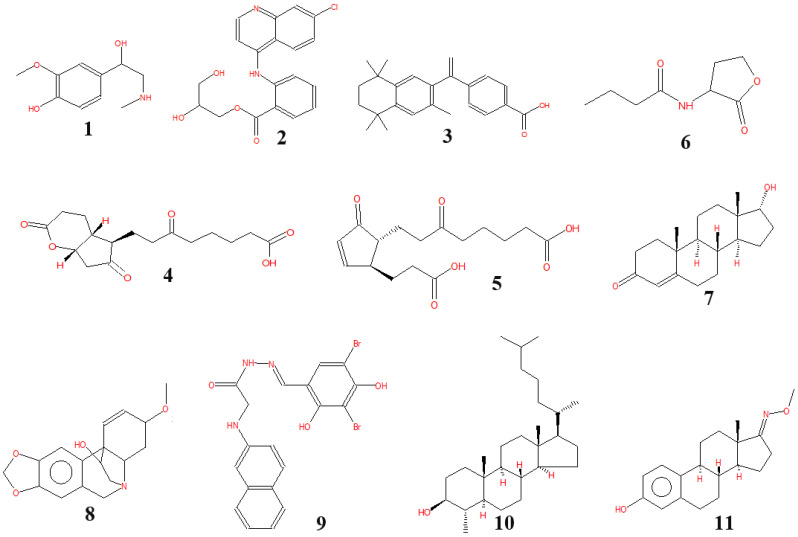
Chemical structures of the volatile compounds (**1–11**) biosynthesized by *P. polymyxa* EB.KN35.

VOCs produced by microorganisms have been described as having the ability to inhibit the growth of bacteria and fungi [46,47,48,53,54,55,56]. VOCs produced by *P. polymyxa* were detected and identified using GC–MS, and the results are shown in Table 2. *P. polymyxa* was found to produce 11 VOCs. Of these, glycine, N-2-naphthalenyl-, 2-[(3,5-dibromo-2,4-dihydroxyphenyl)methylene]hydrazide (**9**) was found in the most abundant amount with an area of 77.58%, metanephrine (**1**) was found to be present in a small amount (0.21%), equal to bexarotene (**3**). Meanwhile, glafenin (**2**) was found to produce an amount twice that of metanephrine (**1**). By contrast, cholestan-3-ol, 4-methyl-, (3β,4α,5α)-(10) and estra-1,3,5(10)-trien-17-one, 3-hydroxy-, O-methyloxime (CAS) (**11**) were produced in moderate amounts (5.01–9.87% of the area), while the remaining VOCs (**4**, **5**, **6**, **7**, and **8**) were found as minor compounds in the culture medium fermented by *P. polymyxa* EB.KN35. Of these, some VOCs, such as **3** and **9**, have been shown to be potential fungicidal compounds [57]. The VOCs produced by *P. polymyxa* were also found to be effective against some nematodes [58,59,60]. *P. polymyxa* J2-4 also produced 14 VOCs, which exhibited nematicidal activity *Meloidogyne incognita* [59]. *P. polymyxa* Sb3-1 created 2-nonanone and 3-hydroxy-2-butanone against *Verticillium longisporum* [60]. The chemical structures of these VOCs are presented in Figure 7.

### 3.5. Molecular Docking and DFT Analysis

Up to date, several studies have reported the potential effect of *P. polymyxa* against *F. oxysporum* [17,18,19,20,21,22,23,24]; however, exploring the mechanism action of *P. polymyxa*’s bioactive compounds against *F. oxysporum* via a docking study has not been reported. In this study, we conducted molecular docking to investigate the interaction and binding energy of the identified compounds produced by *P. polymyxa* toward the protein 1TRY targeting *F. oxysporum.* 1TRY is a crucial metabolic enzyme involved in glycolysis and gluconeogenesis. In *Fusarium oxysporum*, 1TRY is an important enzyme with multiple functions. It is a key metabolic enzyme that also contributes to fungal virulence.

The structure of *F. oxysporum* 1TRY was investigated using a locally developed image plate scanner and reported by Rypniewski et al. (1995) [36]. A single crystal of 1TRY was analyzed on the X31 beamline at EMBL, Hamburg. The final protein model was found to consist of 1557 protein atoms, 400 water molecules, 1 molecule of isopropanol, and 1 molecule of an isopropyl phosphoryl inhibitor group, covalently bound to the catalytic Ser195. There are some fundamental differences in the structures of *F. oxysporum* 1TRY compared to mammalian and *Streptomyces* trypsins. The specificity pocket of *F. oxysporum* 1TRY is larger than in bovine trypsin, which results in the preference of *F. oxysporum* trypsin for the bulkier arginine over lysine. The capacity for binding on the C-terminal side of the substrate is more restricted in *F. oxysporum* trypsin than in mammalian and *Streptomyces griseus* trypsins, leading to the relative inactivation of *F. oxysporum* trypsin toward peptide-pNA substrate analogs.

It has been reported that the possible mechanism of action of a bacterial strain inhibits *F. oxysporum* by producing plant growth-promoting compounds [61], producing fungal cell wall-degrading enzymes [20,21,22,23,24], enhancing the effect of some enzymes, such as catalase, phenylalanine ammonia-lyase, superoxide dismutase, and peroxidase, in plant roots [62], and producing siderophores [63] and antifungal lipopeptides [61]. It has also been evidenced that the anti-*F. oxysporum* effect of bacterial strains is associated with the synthesis of VOCs [19,64]. However, the interaction mechanism of major VOCs has not been reported. Thus, we identified the VOCs produced by *P. polymyxa* EB.KN35 and elucidated the interaction mechanism of these identified VOCs toward the protein 1TRY targeting *F. oxysporum* in this work.

**Figure 8:**
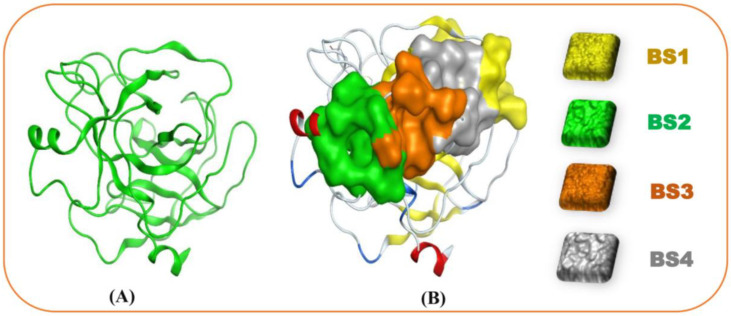
The 3D structure (**A**) and four binding sites (**B**) of 1TRY targeting *F. oxysporum*.

The structural data of 1TRY were obtained from the RCSB Protein Data Bank, its 3D structure was optimized using the MOE software (Figure 8A), and the possible binding sites (BS) of 1TRY were searched using the site finder function of MOE. Four BS were found on this protein 1TRY (Figure 8B), and detailed information on the size and residues of these BS is presented in Table 3. The ligands (11 VOCs identified in this work and asparagine—a commercial 1TRY inhibitor) were docked into all binding sites of the protein 1TRY; for each compound, only the highest interaction was collected as data and described. In molecular docking studies, RMSD and docking score (DS) have been widely used for checking the potential successful interaction of a ligand with its target protein. When a ligand binds to its target protein with an RMSD value under 2.0Å and a DS value under −3.20 kcal/mol, this interaction is accepted, and this compound may be suggested as a possible inhibitor [65].

**Figure 9:**
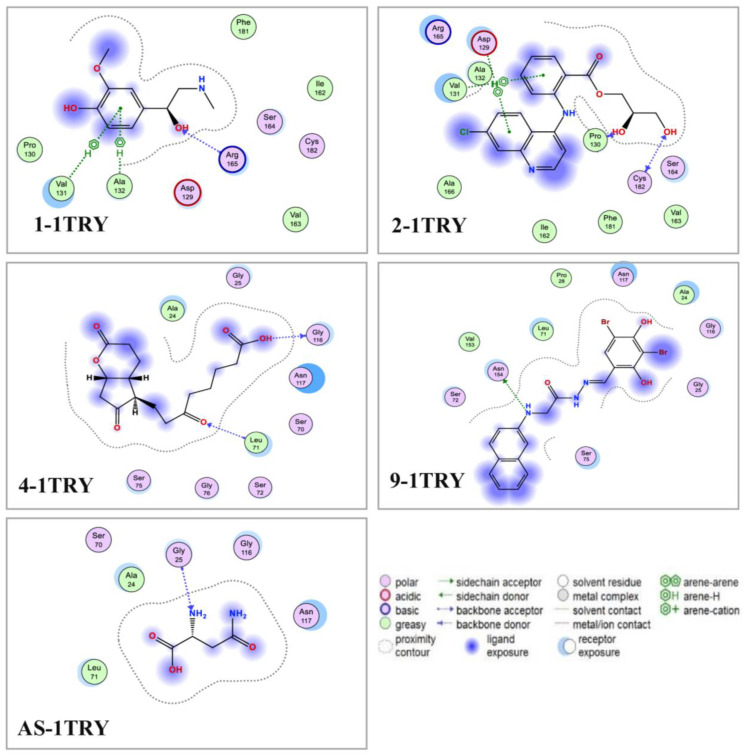
The detailed interaction of the most active ligands (VOCs **1**, **2**, **4**, and **9**) produced by *P. polymyxa* EB.KN35 and asparagine (AS) at the binding sites of 1TRY targeting *F. oxysporum*.

As shown in Table 4, all the checked ligands were found to be interacting with 1TRY with RMSD values in the accepted range (0.87–1.95Å) and, as such, were eligible for further investigation. In addition, these ligands bound to 1TRY with DS values in the range from −7.6 to −10.6 kcal/mol, suggesting that these compounds may be potential inhibitors of the protein 1TRY targeting *F. oxysporum.* In a comparison of DS values, including the positive inhibitor asparagine (AS), the effective binding ability of these ligands toward 1TRY was ranked as follows: **9** > **2** > **1** > **4** ≥ AS (commercial TRY inhibitor) > **5** > **3** > **7** > **8** > **6** > **11**. Based on the comparison of DS values, VOC 4 showed a comparable DS value to AS, while VOCs **1**, **2**, and **9** demonstrated a more efficient interaction with 1TRY than that of the positive 1TRY inhibitor. In addition, VOC 9 was produced by *P. polymyxa* EB.KN35 (Table 2) with the highest productivity, occupying an area of 77.58%, indicating that the strong anti-*F. oxysporum* activity of *P. polymyxa* EB.KN35 may be due to secreting a high content of VOC 9. For a deeper understanding of the interaction of the most active ligands (VOCs **1**, **2**, **4**, and **9**) with protein 1TRY, the detailed interaction of these VOCs with 1TRY at its binding sites is shown in Figure 9.

VOC 1 and 2 are effectively bound to 1TRY at binding site 2 with low DS values of −9.5 and −9.6 kcal/mol, respectively. Among these, VOC 1 interacted with 1TRY via four linkages (1 H-acceptor, three pi-H) by binding to amino acids Arg165 and Ala132 to generate 1 H-acceptor and one pi-H linkage and binding to Val131 to create two pi-H linkages. VOC 2 was found to bind to 1TRY by interacting with amino acids Pro130, Cys182, Asp129, and Val131 to create up to five linkages (2 H-donor, 1 H-acceptor, and two pi-H). Meanwhile, VOC 4, VOC 9, and AS were found to be strongly bound to 1TRY at binding site 1 with low DS values of −9.4, −10.6, and −9.4 kcal/mol, respectively. Among these, AS was only bound to 1TRY through an interaction with one amino acid, Gly25, and one H-acceptor linkage was formed. VOC 4 interacted with 1TRY via linking to Gly116 and Leu71, forming 1 H-donor and 1 H-acceptor, respectively. Similar to AS, VOC 9 was also found to effectively interact with the target protein through one H-donor linkage formed between the ligand and amino acid Asn154.

Trypsin-like proteases such as *F. oxysporum* 1TRY are key virulence factors that degrade plant defense proteins, facilitate invasion, and support fungal growth. Thus, inhibiting these enzymes or genetic modifications may provide potential strategies for controlling *F. oxysporum* [37,38]. In this study, VOCs **1**, **2**, **4**, and **9** showed effective binding to *F. oxysporum* 1TRY. Thus, they may be suggested as potential 1TRY inhibitors, as such, having promising use as control agents of *F. oxysporum*. However, these results were obtained based on screening in silico studies. Thus, further screening tests in vitro for these VOCs need to be conducted for confirmation. In a previous report, compounds such as VOC bexarotene and VOC glycine, N-2-naphthalenyl-, 2-[(3,5-dibromo-2,4-dihydroxyphenyl)methylene]hydrazide, identified as VOCs **3** and **9**, respectively, in this study, were recognized as potential fungicidal compounds via in silico docking studies against protein 3QPC targeting *F. solani* [57]. However, no data have reported the fungicidal effect of the identified compounds against *F. oxysporum*. Thus, this result may indicate a novel potential fungicidal effect of VOCs **1**, **2**, **4**, and **9** against *F. oxysporum* via molecular docking elucidation.

**Figure 10:**
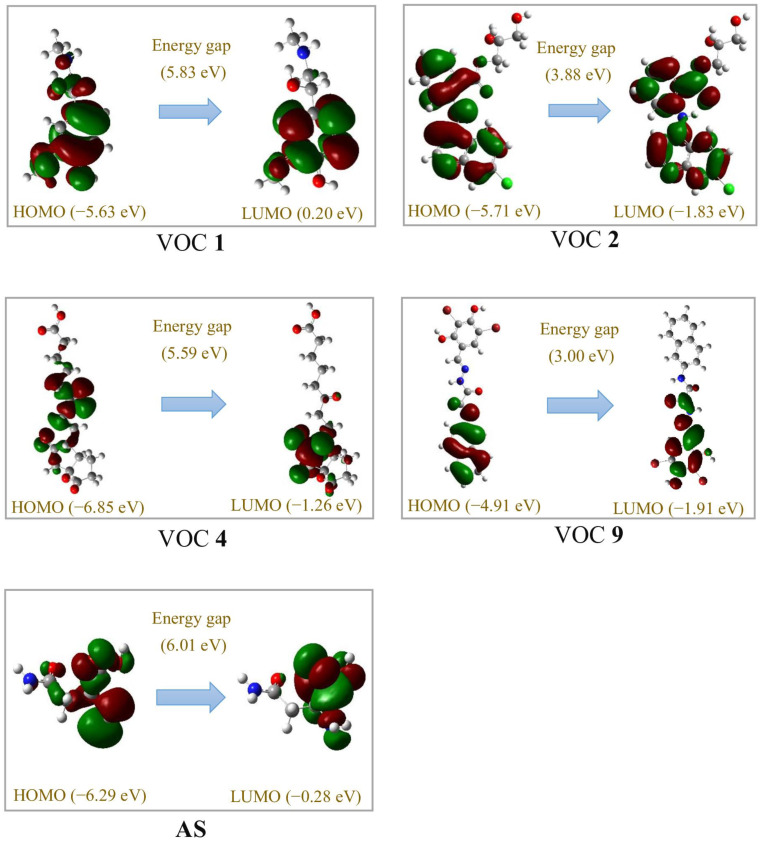
HOMO and LUMO of the most active ligands (VOCs **1**, **2**, **4**, and **9**) produced by *P. polymyxa* EB.KN35 and asparagine (**AS**) analyzed using DFT at the theoretical level of B3LYP/6–31G.

Some of the most potent compounds (VOCs **1**, **2**, **4**, and **9**) and AS were further checked for their highest-energy molecular orbital (HOMO) and the lowest unoccupied molecular orbital (LUMO) using density functional theory (DFT) at the B3LYP/6-31G theoretical level. The data are presented in Figure 10. The structures of these ligands possess low E_Homo_ values (from 4.91 to −6.85 eV), indicating these molecules have significant electronic stability (commonly accepted when the E_Homo_ value is under −5 eV) [65]. In this investigation, all the checked ligands showed an energy gap in the range of 3.00 eV to 6.01 eV. As such, almost all of these compounds have potential intermolecular binding capability to the target protein.

### 3.6. Implications and Recommendations

This study contains some significant new findings and recommendations.

In this study, the durian endophytic *P. polymyxa* EB.KN35 is recorded as being effective against *F. oxysporum* (79.58%) for the first time. This finding broadens the application of *P. polymyxa* as a potential source of biofungicides against the durian pathogenic fungal strain *F. oxysporum*. In addition, EB.KN35 was also found to be secreting some plant growth-promoting compounds such as GA3, IAA, kinetin, and zeatin. As such, this endophytic bacterium could be used as a biofertilizer. However, further investigation is needed, such as evaluating the potential antifungal activity, plant growth-promoting effect, as well as the potential impact on microbial soil systems of *P. polymyxa* EB.KN35 in greenhouse tests and field cultivations for developing this endophytic bacterial strain as an applicable biocontrol agent.

Regarding eco-friendly and effective EB.KN35 biomass production, some agricultural processing byproducts were used as C/N sources for fermentation. This study is the first to report the reuse of a soybean processing byproduct as a novel, low-cost, and friendly substrate for producing *P. polymyxa* via fermentation. The utilization of a 14 L bioreactor system for *P. polymyxa* EB.KN35 fermentation achieved higher biomass productivity in a shorter fermentation time. To achieve higher biomass productivity, optimal fermentation in a bioreactor should be further investigated, and large-scale fermentation may be performed.

The possible mechanism of action of the VOCs produced by *P. polymyxa* against *F. oxysporum* was also explored for the first time via docking with the protein 1TRY targeting anti-pathogenic fungi. The molecular docking results indicated that some VOCs (**1**, **2**, **4**, and **9**) may play a significant role in inhibiting *F. oxysporum*. This result suggested that these VOCs may be potential fungicidal agents for further investigation.

## 4. Conclusions

*P. polymyxa* EB.KN35 was screened as having the most effective fungicidal effect against *F. oxysporum*, and optimal fermentation conditions for its biomass production were investigated. Soybean processing byproducts were newly found to be a suitable substrate for *P. polymyxa* EB.KN35 fermentation. In addition, this bacterial strain was also successfully fermented in a 14 L bioreactor system and achieved a high biomass yield (3.46 × 10^11^ CFU/mL) in a short fermentation time (8 h). This study also demonstrated that *P. polymyxa* EB.KN35 secretes several plant growth-promoting compounds (GA3, IAA, kinetin, and zeatin) and 11 VOCs. Furthermore, based on the molecular docking analysis, some VOCs (**1**, **2**, **4**, and **9**) were suggested as bioactive compounds playing an important role in inhibiting *F. oxysporum.* This study demonstrates the feasibility of using soybean processing byproducts as a cost-effective C/N source for *P. polymyxa* EB.KN35 fermentation, facilitating sustainable biocontrol of *F. oxysporum*. Future research should explore field-scale applications and optimize fermentation parameters for commercial production.

## Figures and Tables

**Table 1:** Antifungal potential of *P. polymyxa* against *F.* oxysporum in crops.

Strain	Origin	Biocontrol	Antifungal Effect (%)	Ref.
*P. polymyxa* EB.KN35	Root of the durian plant	*F. oxysporum*	79.58	This study
*P. polymyxa* SQR-21	Rhizosphere of healthy watermelon plants	*F. oxysporum*	70	[17]
*P. polymyxa* CF05	*Cryptomeria fortunei*	*F. oxysporum* f. sp. *lycoersici*	78.24	[18]
*P. polymyxa* WR-2	-	*F. oxysporum* f. sp. *niveum*	36–40	[19]
*P. polymyxa* NSY50	Vinegar waste compost	*F. oxysporum* f. sp. *cucumerinum*		[20]
*P. polymyxa* SR19	Root of the *Urtica dioica* plant	*F. oxysporum*	≥60	[21]
*P. polymyxa* HX-140	Rhizosphere soil of rape	*F. oxysporum* f. sp. *cucumerinum*	55.6	[22]
*P. polymyxa* hg18	Healthy cucumber plant	*F. oxysporum* f. sp. *cucumerinum*	69.57	[23]
*P. polymyxa* PJH16	Cucumber soil	*F. oxysporum* f. sp. *cucumerinum Foc* FJH36	88.36	[24]

**Table 2:** Identification of the volatile compounds produced by *P. polymyxa* EB.KN35 using GC–MS.

No.	RT (min)	Area (%)	Compounds
1	5.23	0.21	Metanephrine
2	7.94	0.48	Glafenin
3	11.22	0.2	Bexarotene
4	26.01	0.05	Cyclopenta[b]pyran-5-octanoic acid, octahydro-ε,2,6-trioxo-, (4aR,5R,7aS)-
5	26.71	0.05	Tetranorprostaglandin J2
6	28.71	0.05	N-butyryl-DL-homoserine lactone
7	36.66	0.04	Epitestosterone
8	38.17	0.03	Crinan-11-ol, 1,2-didehydro-3-methoxy-, (3β,5α,11S,13β,19α)- (CAS)
9	38.67	77.58	Glycine, N-2-naphthalenyl-, 2-[(3,5-dibromo-2,4-dihydroxyphenyl)methylene]hydrazide
10	39.59	9.87	Cholestan-3-ol, 4-methyl-, (3β,4α,5α)-
11	44.62	5.01	Estra-1,3,5(10)-trien-17-one, 3-hydroxy-, O-methyloxime (CAS)

**Table 3:** Sizes and residues of the binding sites on the protein 1TRY.

Site No.	Size	Residues
1	27	Ala24, Gly25, Asp26, Phe27, Pro28, Ser70, Leu71, Ser72, Gly116, Asn117, Asn154
2	24	Asp129, Pro130, Val131, Ala132, Ile162, Val163, Ser164, Arg165, Cys168, Met180, Phe181, Cys182,
3	16	Pro130, Val131, Ser134, Ser135, Ala136, Ile162, Asp201, Ser201a, Ile210
4	15	Asp26, Phe27, Pro28, Phe29, Thr137, Lys157, Val200, Asp201, Asn203

**Table 4:** Docking study data of the docking of ligands (L, volatile compounds) and asparagine (AS) binding with the protein (P) 1TRY targeting *F. oxysporum*.

Ligands	Symbol of the L–P Complex	Binding Site	RMSD (Å)	DS (kcal/mol)	Linkages	Interactions (Distance (Å)/E (kcal/mol) / Linkage Type)
1	1-1TRY	2	1.36	−9.5	4 (1 H-acceptor, 3 pi-H)	Arg165 (3.06/−2.4/H-acceptor) Val131 (4.44/−0.6/pi-H) Val131 (4.69/−1.0/pi-H) Ala132 (4.69/−1.0/pi-H)
2	2-1TRY	2	1.82	−9.6	5 (2 H-donor, 1 H-acceptor, 2 pi-H)	Pro130 (3.32/−1.0/H-donor) Cys182 (3.14/−1.1/H-donor) Cys182 (3.21/−0.4/H-acceptor) Asp129 (4.46/−0.8/pi-H) Val131 (4.78/−0.6/pi-H)
3	3-1TRY	1	1.21	−9.0	2 (1 H-donor, 1 pi-H)	Ser70 (2.96/−0.9/H-donor) Ser75 (3.96/−1.2/pi-H)
4	4-1TRY	1	0.87	−9.4	2 (1 H-donor, 1 H-acceptor)	Gly116 (3.04/−1.5/H-donor) Leu71 (3.21/−2.0/H-acceptor)
5	5-1TRY	2	1.80	−9.1	5 (3 H-donor, 2 H-acceptor)	Asp129 (3.04/−1.4/H-donor) Pro130 (2.83/−1.3/H-donor) Asp129 (3.12/−5.0/H-donor) Ala132 (3.29/−0.6/H-acceptor) Arg165 (2.95/−3.1/H-acceptor)
6	6-1TRY	2	1.86	−7.86	2 (1 H-donor, 1 pi-H)	Asp129 (2.94/−4.3/H-donor) Ala132 (3.02/−2.9/H-acceptor)
7	7-1TRY	2	1.66	−8.7	1 H-donor	Try120 (3.37/−0.5/H-donor)
8	8-1TRY	1	1.90	−8.5	1 H-donor	Asn117 (2.95/−0.6/H-donor)
9	9-1TRY	1	1.95	−10.6	1 H-donor	Asn154 (3.42/−0.8/H-donor)
10	10-1TRY	4	1.86	−8.2	1 H-donor	Asp26 (3.13/−0.6/H-donor)
11	11-1TRY	4	1.47	−7.6	H-pi	Phe27 (4.21/−0.7/H-pi)
AS	AS-1TRY	1	1.81	−9.4	1 H-acceptor	Gly25 (3.15/−1.9/H-acceptor)

## Data Availability

The original contributions presented in the study are included in the article; further inquiries can be directed to the corresponding authors.

## Appendix A

**Table A1:** Evaluation of the antifungal potential of endophytic bacteria against pathogenic fungus *F. oxysporum*.

No.	Strain	Antifungal Activity (%)	No.	Strain	Antifungal Activity (%)
** 1 **	EB.CK1	-	56	EB.KN27	17.44
** 2 **	EB.CK2	48.72	57	EB.KN28	48.72
** 3 **	EB.CK3	-	58	EB.KN29	-
** 4 **	EB.CK4	-	59	EB.KN30	-
** 5 **	EB.CK5	28.21	60	EB.KN31	28.97
** 6 **	EB.CK6	-	61	EB.KN32	-
** 7 **	EB.CK7	10.26	62	EB.KN33	34.87
** 8 **	EB.CK8	-	63	EB.KN34	-
** 9 **	EB.CK9	50.83	64	EB.KN35	79.58
** 10 **	EB.CK10	-	65	EB.KN36	-
** 11 **	EB.CK11	43.59	66	EB.KN37	-
** 12 **	EB.CK12	-	67	EB.KN38	32.31
** 13 **	EB.CK13	27.69	68	EB.KN39	-
** 14 **	EB.CK14	48.97	69	EB.KN40	-
** 15 **	EB.CK15	-	70	EB.KN41	48.97
** 16 **	EB.CK16	-	71	EB.KN42	-
** 17 **	EB.CK17	-	72	EB.KN43	48.46
** 18 **	EB.CK18	42.31	73	EB.KN44	-
** 19 **	EB.CK19	47.69	74	EB.KN45	11.79
** 20 **	EB.CK20	-	75	EB.KN46	-
** 21 **	EB.CK21	48.72	76	EB.KN47	-
** 22 **	EB.CK22	23.59	77	EB.EH1	42.31
** 23 **	EB.CK23	-	78	EB.EH2	41.79
** 24 **	EB.CK24	-	79	EB.EH3	-
** 25 **	EB.CK25	39.74	80	EB.EH4	23.59
** 26 **	EB.CK26	34.62	81	EB.EH5	-
** 27 **	EB.CK27	-	82	EB.EH6	40.77
** 28 **	EB.CK28	-	83	EB.EH7	46.92
** 29 **	EB.CK29	32.05	84	EB.EH8	-
** 30 **	EB.KN1	21.28	85	EB.EH9	-
** 31 **	EB.KN2	-	86	EB.EH10	-
** 32 **	EB.KN3	-	87	EB.EH11	-
** 33 **	EB.KN4	48.72	88	EB.EH12	44.36
** 34 **	EB.KN5	41.28	89	EB.EH13	-
** 35 **	EB.KN6	-	90	EB.EH14	-
** 36 **	EB.KN7	-	91	EB.EH15	19.49
** 37 **	EB.KN8	8.97	92	EB.EH16	-
** 38 **	EB.KN9	-	93	EB.EH17	45.90
** 39 **	EB.KN10	51.25	94	EB.EH18	56.67
** 40 **	EB.KN11	37.44	95	EB.EH19	48.97
** 41 **	EB.KN12	31.28	96	EB.EH20	-
** 42 **	EB.KN13	-	97	EB.EH21	49.23
** 43 **	EB.KN14	48.72	98	EB.EH22	-
** 44 **	EB.KN15	48.97	99	EB.EH23	-
** 45 **	EB.KN16	-	100	EB.EH24	-
** 46 **	EB.KN17	16.92	101	EB.EH25	37.44
** 47 **	EB.KN18	-	102	EB.EH26	-
** 48 **	EB.KN19	36.67	103	EB.EH27	-
** 49 **	EB.KN20	-	104	EB.EH28	22.82
** 50 **	EB.KN21	-	105	EB.EH29	-
** 51 **	EB.KN22	-	106	EB.EH30	-
** 52 **	EB.KN23	-	107	EB.EH31	-
** 53 **	EB.KN24	49.23	108	EB.EH32	-
** 54 **	EB.KN25	31.28	109	EB.EH33	48.46
** 55 **	EB.KN26	-	110	EB.EH34	55.83
